# Socioeconomic and Demographic Characteristics of US Adults Who Purchase Prescription Drugs From Other Countries

**DOI:** 10.1001/jamanetworkopen.2020.8968

**Published:** 2020-06-24

**Authors:** Young-Rock Hong, Juan M. Hincapie-Castillo, Zhigang Xie, Richard Segal, Arch G. Mainous

**Affiliations:** 1 Department of Health Services Research Management, and Policy, College of Public Health and Health Professions, University of Florida, Gainesville; 2 Department of Pharmaceutical Outcomes and Policy, College of Pharmacy, University of Florida, Gainesville; 3 Center for Drug Evaluation and Safety, University of Florida, Gainesville; 4 Department of Community Health and Family Medicine, College of Medicine, University of Florida, Gainesville

## Abstract

**Importance:**

Little is known about the current use of imported drugs and the factors associated with individual purchase of medications outside the US.

**Objective:**

To evaluate the proportion of the US adult population that purchases medications in other countries and the patient factors associated with the behavior.

**Design, Setting, and Participants:**

This retrospective cross-sectional study used data from the 2015-2017 National Health Interview Survey. The study sample included 61 238 individuals 18 years or older who reported use of prescribed medication by a physician or other practitioner. Data analysis was performed in November 2019.

**Main Outcomes and Measures:**

Self-reported experience of purchasing prescription drugs from countries outside the US in the past 12 months. Internet use behaviors for health care included searches for health information and filling of a prescription online. Medication-taking behaviors included skipping or delaying filling a prescription and using alternative therapies to save money. Survey design–adjusted analysis was used to estimate and compare characteristics between those who purchased medications outside the US and those did not. Multivariable logistic regression was fitted to examine the association of medication purchases with internet use and medication-taking behavior factors.

**Results:**

Among 61 238 US adults taking prescription medications (mean [SD] age, 50.5 [18.5] years; 56.5% female; 70.8% white), the estimated prevalence of purchasing of medication outside the US was 1.5% (95% CI, 1.4%-1.7%; 2.3 million US individuals). Those who purchased medications outside the US were more likely to be older (age >64 years; adjusted odds ratio [aOR], 1.68; 95% CI, 1.24-2.29), to be from Hispanic (aOR, 1.70; 95% CI, 1.23-2.35) or immigrant populations (aOR, 3.20; 95% CI, 2.44-4.20), and to have higher educational attainment (bachelor's degree; aOR, 1.79; 95% CI, 1.27-2.54), lower family income (low income; aOR, 1.41; 95% CI, 1.06-1.87), and lack of insurance (aOR, 3.14; 95% CI, 2.33-4.21). Data analyses indicated that online health information–seeking behavior (aOR, 1.62; 95% CI, 1.33-1.98) or use of an online pharmacy (aOR, 2.30; 95% CI, 1.83-2.90) was associated with a greater likelihood of medication purchases outside the US. Individuals who skipped medications (aOR, 3.86; 95% CI, 3.05-4.88) or delayed filling a prescription (aOR, 4.04, 95% CI, 3.23-5.06) also had higher odds of purchasing medication outside the US.

**Conclusions and Relevance:**

The findings suggest that patients are not using prescription purchases outside the US to meet their medication needs. However, monitoring to promote safe administration of medications imported into the US should be continued.

## Key Points

**Question:**

How many US adults buy or import drugs to save money, and what factors are associated with their purchase of medications outside the US?

**Findings:**

In this cross-sectional study of 61 238 US adults taking prescription medication, 1.5% reported medication purchases from countries outside the US. Use of the internet for health information and online pharmacy purchases was associated with medication purchases outside the US.

**Meaning:**

The findings suggest that patients are not using prescription purchases outside the US to meet their medication needs.

## Teaser

This cross-sectional study evaluates the proportion of US adults who purchase prescription medications in other countries and the patient factors associated with the behavior.

## Introduction

Health care spending in the US is substantially higher than in other high-income nations and has outpaced overall national economic growth for several decades.^1^ US health care spending now reaches $3.65 trillion, accounting for 17.7% of the gross domestic product in 2018.^2^ According to the US Government Accountability Office, US spending on drugs increased by approximately 25% from 2013 and 2017.^3^ Recent estimates suggest that overall out-of-pocket costs have increased from $56 billion in 2014 to $61 billion in 2018.^4^ Furthermore, forecasts suggest that the net spending in medications will increase from $344 billion in 2018 to $420 billion by 2023.^4^

As patients are faced with decisions on how to purchase these medications, a multitude of proposals at the state and federal level have been forwarded as strategies to counteract the increasing drug pricing issues.^5,6,7^ Because drug prices in some other countries, especially Canada, are lower than those in the US, the Trump administration plans to allow drug importation—also known as the Safe Importation Action Plan—from Canada and possibly other countries to stimulate price competition.^8^ The US Food and Drug Administration is also taking steps to establish this pathway and others for safe drug importation in compliance with §801 of the Federal Food, Drug, and Cosmetic Act.^9^

When devising policies to address drug importation successfully, policy makers should understand the factors associated with recent individual purchase of medications outside the US. Previous work^10^ has suggested that financial difficulties associated with being uninsured or underinsured are the main factors associated with this pattern; however, little is known about the characteristics and behavioral patterns (eg, internet use and medication-taking behaviors) of individuals who purchased their medications outside the US. Cost-related medication nonadherence (ie, skipping doses or delaying prescription refill because of cost) is a significant problem in chronic disease management.^11,12^ It is not clear whether medication purchases outside the US to save money are associated with medication adherence. Moreover, internet access makes it easier for many US adults to buy cheaper drugs or refill their prescriptions online. Currently, there is a gap in the literature on the characteristics of patients who buy prescription medicine outside the US. To address these research gaps, this study aimed to use nationally representative survey data (1) to evaluate the proportion of the US adult population who recently purchased prescription medications outside the US to save money, (2) to identify the key socioeconomic and patient factors associated with purchasing medications outside the US, and (3) to assess the associations between internet use and medication-taking behaviors and medication purchases outside the US.

## Methods

### Data Source and Study Sample

This retrospective, cross-sectional study used aggregated data from the 2015-2017 National Health Interview Survey (NHIS). The NHIS is a nationally representative survey of the civilian, noninstitutionalized US population with a mean response rate of 80% that is conducted annually by the National Center for Health Statistics to track health status and health services use in the US.^13^ The NHIS is the country’s largest in-person household health survey that has been used extensively in health services research to address medication-related issues of cost and nonadherence. To include both household and individual-level information, we merged 3 NHIS components (adult sample, person, and family files) using a unique person identifier. This study was deemed exempt from review by the University of Florida Institutional Review Board because we used publicly available, deidentified data; therefore, no informed consent was required. The study followed the Strengthening the Reporting of Observational Studies in Epidemiology (STROBE) reporting guideline.^14^

We analyzed data for survey respondents 18 years or older who reported taking medication prescribed by a physician or other health care professional in the past 12 months. We excluded respondents who had missing information on socioeconomic variables, health insurance status, and health information use (<0.5% missingness). eTable 1 in the Supplement presents the steps of the analytic sample selection process.

### Measures

#### Medication Purchases Outside the US

Our primary outcome variable was the purchase of prescription drugs from countries outside the US. Survey respondents were asked whether they bought prescription drugs from a country other than the US to save money during the past 12 months. We defined respondents as purchasers of medications outside the US if they answered yes to the question.

#### Internet Use Behaviors

Internet use behaviors for health care were measured with 3 items: (1) searching for health information on the internet, (2) using online chat groups to learn about health topics, and (3) filling a prescription online during the past 12 months. We constructed binary variables of each item (yes vs no).

#### Medication-Taking Behaviors

Survey participants were also asked whether they (1) skipped medication doses, (2) delayed filling a prescription, or (3) used alternative therapies to save money in the past 12 months. Each item was dichotomized according to whether a participant reported the experiences.

#### Sociodemographic and Health-Related Covariates

Other covariates that we included were respondents’ age (18-44, 45-64, or ≥65 years of age), sex (male or female), race/ethnicity (non-Hispanic white, non-Hispanic black, Hispanic, or other), immigration status (born in the US or not), marital status (married or unmarried), employment status (employed or unemployed), educational level (less than high school, high school or General Educational Development, some college, bachelor’s degree, or graduate degree or higher), family income (low income [federal poverty level <200%], middle income [federal poverty level of 200%–400%], and high income [federal poverty level >400%]), census region (Northeast, Midwest, South, or West), health insurance (private, public, or uninsured), self-reported general health status (excellent or good vs poor or fair), and the number of comorbidities (0, 1, 2, or ≥3). Comorbidities included reports of physician-diagnosed hypertension, diabetes, cardiovascular disease (including coronary heart diseases, angina, myocardial infarction, and other heart diseases), stroke, emphysema, asthma, chronic obstructive pulmonary disease, ulcer, chronic pain (neck, back, or joint), problems with vision or hearing, or any type of cancer.

### Statistical Analysis

We calculated the weighted prevalence of purchasing medication outside the US to obtain a nationally representative estimate from the combined survey data from January 1, 2015, to December 31, 2017. We followed the analytic guidelines on NHIS data and variance estimation.^15^ Adjusted Wald *F* tests were used to compare characteristics between those who purchased medications outside the US and those who did not. We fitted multivariable logistic regression models to examine the likelihood of medication purchases across individual characteristics, internet use, and medication-taking behavior factors. Sociodemographic and health-related covariates included age, race/ethnicity, immigration status, employment status, educational level, family income, census region, type of insurance, general health status, and the number of comorbidities. We also analyzed the primary outcome in subgroups: racial/ethnic groups by immigration status (eg, US-born white individuals, white immigrants, US-born black individuals, and black immigrants) and insurance types by Medicare eligibility (age ≥65 years) and prescription drug benefit (Medicare Part D). Race/ethnicity and immigration status were self-reported, and we conducted these subgroup analyses to further understand whether certain races/ethnicities with immigration status or more specific insurance types were disproportionately associated with medication purchases outside the US. Lastly, we estimated the prevalence of medication purchases outside the US separately for 5 major chronic conditions (hypertension, diabetes, cardiovascular disease, chronic obstructive pulmonary disease, and cancer). Data analysis was performed in November 2019. All analyses were conducted using the PROC SURVEY procedure in SAS, version 9.4 (SAS Institute Inc) to account for selection probability, oversampling, and nonresponse in the survey.^15^ Statistical significance was tested at a 2-sided *P* < .05.

## Results

### Prevalence of Prescription Medication Purchases Outside the US to Save Money

A total of 61 238 respondents 18 years or older in the NHIS 2015-2017 (mean [SE] age, 50.5 [18.5] years; 56.5% female; 70.8% non-Hispanic white), representing 152.2 million US adults taking prescription medications, were included in this study. The weighted prevalence of purchasing of medications outside the US was 1.5% (95% CI, 1.4%-1.7%; 2.3 million US individuals). Before adjustment, the prevalence of purchasing of medication outside the US was significantly different by age, race/ethnicity, immigration status, marital status, educational level, family income, type of health insurance, and self-reported health status (Table 1). Specifically, individuals who purchased medication outside the US were more likely to be middle-aged (1.3% of those aged 18-44 years vs 1.8% of those aged 45-64 years, *P* = .004), Hispanic (4.2% vs 0.6% non-Hispanic black, *P* < .001), or immigrants (4.4% vs 1.0% of those who were born in the US, *P* < .001) and to have a higher educational level (1.1% of those with some high school or General Educational Development vs 2.1 of those with graduate school or higher, *P* < .001), lower family income (2.0% of those with low income vs 1.3% of those with high income, *P* < .001), lack of insurance (5.0% vs 1.3% of those with private insurance, *P* < .001), or poor or fair reported health status (2.2% vs 1.4% of those with excellent or good reported health status, *P* = .001). The prevalence of purchasing of medication outside the US did not increase with an increasing number of comorbidities.

**Table 1.** Estimated Prevalence and Factors Associated With Medication Purchase Outside the US Among US Adults Taking Prescription Medications by Socioeconomic and Health Characteristics

Characteristic	No./total No. (%)		Unadjusted weighted prevalence, % (95% CI)^a^	*P* value	Adjusted odds ratio (95% CI)^b^	*P* value
	Unweighted	Weighted^a^				
Overall	927/61 238 (1.5)	2 339 900/152 249 180 (1.5)	1.5 (1.4-1.7)	NA	NA	NA
Age group, y						
18-44	250/18 578 (1.3)	686 678/54 474 847 (1.3)	1.3 (1.0-1.5)	.004	1 [Reference]	NA
45-64	361/21 900 (1.6)	1 002 225/57 214 352 (1.8)	1.8 (1.5-2.0)		1.42 (1.12-1.79)	.004
≥65	316/20 760 (1.5)	650 997/40 559 981 (1.6)	1.6 (1.4-1.8)		1.68 (1.24-2.29)	.001
Sex						
Male	384/24 981 (1.5)	1 050 313/66 292 234 (1.6)	1.6 (1.4-1.8)	.52	1 [Reference]	NA
Female	543/36 257 (1.5)	1 289 586/85 956 945 (1.5)	1.5 (1.3-1.7)		0.98 (0.82-1.17)	.81
Race/ethnicity						
Non-Hispanic white	556/44 417 (1.3)	1 291 784/107 752 090 (1.2)	1.2 (1.1-1.3)	<.001	1 [Reference]	NA
Non-Hispanic black	40/7081 (0.6)	95 413/17 307 779 (0.6)	0.6 (0.3-0.8)		0.40 (0.28-0.58)	<.001
Hispanic	276/6414 (4.3)	773 839/18 402 355 (4.2)	4.2 (3.4-5.0)		1.70 (1.23-2.35)	.001
Other	55/3326 (1.7)	178 864/87 869,55 (2.0)	2.0 (1.4-2.7)		0.70 (0.47-1.05)	.09
Immigration status						
US born	593/53 671 (1.1)	1 355 931/129 867 717 (1.0)	1.0 (0.9-1.2)	<.001	1 [Reference]	NA
Immigrant	334/7567 (4.4)	983 968/22 381 462 (4.4)	4.4 (3.8-5.0)		3.20 (2.44-4.20)	<.001
Marital status						
Not married	460/33 650 (1.4)	942 635/67 951 689 (1.4)	1.4 (1.2-1.6)	.05	1 [Reference]	NA
Married	467/27 588 (1.7)	1 397 265/84 297 491 (1.7)	1.7 (1.5-1.9)		1.05 (0.86-1.30)	.62
Employment						
Not employed	492/30 780 (1.6)	1 147 068/69 399 288 (1.7)	1.7 (1.4-1.9)	.11	1 [Reference]	NA
Employed	435/30 458 (1.4)	1 192 831/82 849 892 (1.4)	1.4 (1.2-1.6)		0.94 (0.73-1.22)	.64
Educational level						
Less than school	157/7471 (2.1)	403 058/17 715 534 (2.3)	2.3 (1.7-2.8)	<.001	1 [Reference]	NA
High school or GED	158/14 879 (1.1)	416 044/36 458 051 (1.1)	1.1 (0.9-1.4)		1.02 (0.71-1.47)	.90
Some college	262/19 472 (1.3)	654 108/47 553 147 (1.4)	1.4 (1.1-1.6)		1.51 (1.09-2.11)	.02
Bachelor's degree	181/11 842 (1.5)	465 648/30 983 742 (1.5)	1.5 (1.2-1.8)		1.79 (1.27-2.54)	.001
Graduate degree or higher	169/7574 (2.2)	401 041/19 538 707 (2.1)	2.1 (1.7-2.4)		2.61 (1.80-3.77)	<.001
Family income level^c^						
Low	377/21 587 (1.7)	916 409/46 321 974 (2.0)	2.0 (1.7-2.3)	.001	1.41 (1.06-1.87)	.02
Middle	280/19 518 (1.4)	715 157/49 331 513 (1.4)	1.4 (1.2-1.7)		1.19 (0.93-1.52)	.17
High	270/20 133 (1.3)	708 333/56 595 692 (1.3)	1.3 (1.0-1.5)		1 [Reference]	NA
Census region						
Northeast	115/10 390 (1.1)	322 725/27 716 385 (1.2)	1.2 (0.9-1.4)	<.001	1 [Reference]	NA
Midwest	163/14 158 (1.2)	387 883/35 359 403 (1.1)	1.1 (0.9-1.3)		1.19 (0.88-1.61)	.26
South	350/21 806 (1.6)	878 203/55 692 331 (1.6)	1.6 (1.3-1.9)		1.37 (1.03-1.81)	.03
West	299/14 884 (2.0)	751 088/33 481 061 (2.2)	2.2 (1.9-2.6)		1.70 (1.26-2.28)	.001
Type of insurance						
Any private	351/27 961 (1.3)	1 001 002/79 755 205 (1.3)	1.3 (1.1-1.4)	<.001	1 [Reference]	NA
Any public	405/30 035 (1.3)	922 841/64 119 518 (1.4)	1.4 (1.2-1.6)		0.82 (0.59-1.14)	.24
Uninsured	171/3242 (5.3)	416 057/8 374 456 (5.0)	5.0 (3.9-6.1)		3.14 (2.33-4.21)	<.001
Self-reported health						
Excellent or good	700/49 646 (1.4)	1 767 143/125 773 928 (1.4)	1.4 (1.3-1.6)	.001	1 [Reference]	NA
Poor or fair	227/11 592 (2.0)	572 757/26 475 251 (2.2)	2.2 (1.8-2.6)		1.32 (1.06-1.66)	.02
No. of comorbidities						
0	250/17 501 (1.5)	661 633/48 477 280 (1.4)	1.4 (1.4-1.6)	.18	1 [Reference]	NA
1	274/18 555 (1.5)	726364/47 364 114 (1.5)	1.5 (1.3-1.8)		1.02 (0.82-1.28)	.83
2	193/11 919 (1.5)	448 479/27 778 408 (1.6)	1.6 (1.6-1.9)		1.17 (0.89-1.54)	.25
≥3	210/13 263 (1.7)	503 424/28 629 378 (1.7)	1.7 (1.6-2.1)		1.32 (0.97-1.78)	.07

Abbreviations: FPL, federal poverty level; GED, general educational development; NA, not applicable.

^a^ Estimates are weighted to be nationally representative.

^b^ Adjustments were for age; sex; race/ethnicity; immigration, marital, and employment status; educational level; family income level; census region; type of insurance; self-reported health status; and the number of comorbidities.

^c^ Low is an FPL less than 200%; middle, FPL of 200 to 400; and high, FPL greater than 400%.

### Socioeconomic and Health Characteristics Associated With Medication Purchases Outside the US

In the multivariable logistic regression model, age, race/ethnicity, immigration status, educational level, family income, region, type of insurance, and self-reported health status were significantly associated with medication purchase outside the US (Table 1). After controlling for socioeconomic and health characteristics, those 65 years or older were 1.68 times (adjusted odds ratio [aOR], 1.68; 95% CI, 1.24-2.29) more likely to have purchased medication outside the US compared with those aged 18-44 years. Hispanic individuals (aOR, 1.70; 95% CI, 1.23-2.35) and immigrants (aOR, 3.20; 95% CI, 2.44-4.20) had higher odds of purchasing medication outside the US than non-Hispanic white individuals and those born in the US. Other significant socioeconomic factors for purchasing medication outside the US included having a graduate or higher degree (aOR, 2.61; 95% CI, 1.80-3.77), having low family income (aOR, 1.41; 95% CI, 1.06-1.87), and being uninsured (aOR, 3.14; 95% CI, 2.33-4.21). Those who reported poor or fair health status were 1.32 times (aOR 1.32; 95% CI, 1.06-1.66) more likely to purchase medication outside the US compared with those who reported excellent or good health status.

### Internet Use and Medication Behavior Factors Associated With Medication Purchases Outside the US

Internet use for health care and medication-taking behavior factors were associated with medication purchase outside the US (Table 2). Our analyses indicated that online health information–seeking behaviors were associated with greater likelihood of medication purchases outside the US. Individuals who searched for health information (aOR, 1.62; 95% CI, 1.33-1.98) or used online chat groups to learn about health care (aOR, 2.07; 95% CI, 1.45-2.94) were approximately twice as likely to purchase medication outside the US than those who did not. Those who filled a prescription online also had a greater likelihood of purchasing medication outside the US (aOR, 2.30; 95% CI, 1.83-2.90). Regarding medication-taking factors, those who skipped medications (aOR, 3.86; 95% CI, 3.05-4.88) or delayed filling a prescription (aOR, 4.04, 95% CI, 3.23-5.06) had higher odds of purchasing medication outside the US. Use of alternative therapies rather than medications prescribed was associated with significantly increased odds of purchasing medication outside the US (aOR 9.23; 95% CI, 7.34-11.61).

**Table 2.** Estimated Prevalence and Factors Associated With Medication Purchase Outside the US Among US Adults Taking Prescription Medications by Internet Use and Medication-Taking Behaviors

Characteristic	No./total No. (%)		Unadjusted weighted prevalence, % (95% CI)^a^	*P* value	Adjusted odds ratio (95% CI)^b^	*P* value
	Unweighted	Weighted^a^				
**Internet use for health care**						
Searching for health information						
No	358/28 737 (1.2)	930 140/66 716 179 (1.4)	1.4 (1.2-1.6)	.04	1 [Reference]	NA
Yes	569/32 501 (1.8)	1 409 760/85 533 001 (1.6)	1.6 (1.5-1.8)		1.62 (1.33-1.98)	<.001
Using online chat groups to learn about health topics						
No	855/58 751 (1.5)	2 129 106/145 520 058 (1.5)	1.5 (1.3-1.6)	.001	1 [Reference]	NA
Yes	72/2487 (2.9)	210 794/6 729 121 (3.1)	3.1 (2.2-4.1)		2.07 (1.45-2.94)	<.001
Filing a prescription on the internet						
No	727/53 657 (1.4)	1 816 290/131 647 289 (1.4)	1.4 (1.2-1.5)	<.001	1 [Reference]	NA
Yes	200/7581 (2.6)	523 610/20 601 891 (2.5)	2.5 (2.1-3.0)		2.30 (1.83-2.90)	<.001
**Medication-taking behaviors**						
Skipped medication doses						
No	721/57 485 (1.3)	1 852 474/143 264 671 (1.3)	1.3 (1.2-1.4)	<.001	1 [Reference]	NA
Yes	206/3753 (5.5)	487 425/8 984 509 (5.4)	5.4 (4.4-6.4)		3.86 (3.05-4.88)	<.001
Delayed filling a prescription						
No	687/56 338 (1.2)	1 754 579/140 508 269 (1.2)	1.2 (1.12-1.38)	<.001	1 [Reference]	NA
Yes	240/4900 (4.9)	585 321/11 740 911 (5.0)	5.0 (4.1-5.9)		4.04 (3.23-5.06)	<.001
Used alternative therapies						
No	676/58 559 (1.2)	1 674 638/145 717 468 (1.1)	1.1 (1.0-1.3)	<.001	1 [Reference]	NA
Yes	251/2679 (9.4)	665 262/6 531 711 (10.2)	10.2 (8.4-12.0)		9.23 (7.34-11.61)	<.001

Abbreviation: NA, not applicable.

^a^ Estimates are weighted to be nationally representative.

^b^ Adjustments were for age; sex; race/ethnicity; immigration, marital, and employment status; educational level; family income level; census region; type of insurance; self-reported health status; and the number of comorbidities.

### Subgroup Analyses

We used the same multivariable logistic regression models to calculate the prevalence of purchasing medication outside the US with more granular estimates by immigration status and race/ethnicity (Figure, A), age and type of insurance (Figure, B), and selected health conditions (Figure, C). The racial/ethnic distribution of individuals who purchased medication outside the US was similar between those who were born in the US and immigrants, with the highest estimates for Hispanic individuals (2.9% of US born and 6.4% of immigrants) (eTable 2 in the Supplement). Being an immigrant was associated with a 1.3% to 3.5% increase in estimates across racial/ethnic groups. Among individuals aged 18 to 64 years, those without insurance had the highest estimates (4.5%) of purchasing medication outside the US, followed by those with marketplace insurance (3.4%), employer-based insurance (1.5%), and Medicaid or other public insurance (1.1%). The probability for purchasing medication outside the US varied little by type of health insurance among those ≥65 years or older, with the highest estimate for those with Medicare (3.7%). There was no significant difference in estimates overall for individuals with Medicare Part D. The estimates also did not significantly differ across selected chronic conditions.

**Figure.**
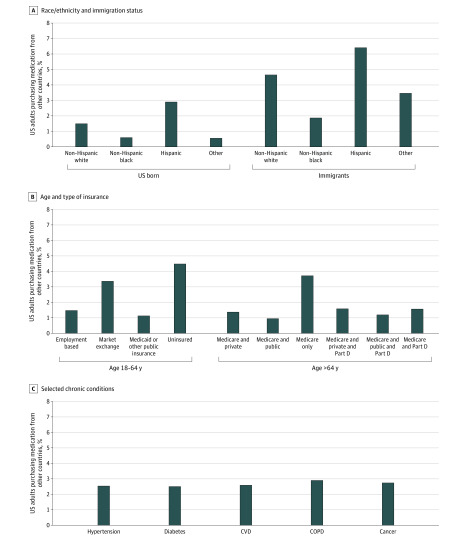
Predicted Estimates of Medication Purchase Outside the US Predicted estimates were generated from the multivariable logistic regression models including age, sex, race/ethnicity, immigration status, marital status, employment status, educational level, family income level, census region, type of insurance, self-reported health status, and the number of comorbidities. COPD indicates chronic obstructive pulmonary disease; CVD, cardiovascular disease.

## Discussion

In this nationally representative sample of US adults regarding recent purchase of prescription medication outside the US to save money, the estimated prevalence of medication purchases outside the US was 1.5%, representing 2.3 million US adults. Although the overall prevalence is relatively low for this behavior, this amount represents several million people. Patients did not seem to be using prescription purchases outside the US to meet their medication needs. These nationally representative data on recent behavior are consistent with other US government estimates but inconsistent with estimates from the Kaiser Family Foundation.^16,17^ The current estimate is lower than the estimate of drug importation reported by the Kaiser Family Foundation (approximately 19 million US adults), which was calculated with the simple extrapolation to the US population in 2016.^16,17^ Our population estimate of prescription medication purchases from other countries may be more accurate given a larger sample size (61 238 vs 1202) and the established representativeness of the NHIS data.^13^

Among sociodemographic factors, we found that older age, being Hispanic or an immigrant, having higher educational attainment, having lower family income, and being uninsured were independently associated with purchasing of medications outside the US. Consistent with previous studies,^12,18^ disadvantaged sociodemographic characteristics (eg, racial/ethnic minority group and lower income) associated with cost-related medication adherence were also found to be factors associated with medication purchase outside the US to reduce the cost burden. However, having more comorbidities was not associated with the medication purchase outside the US as a means of saving costs. Patients with multiple conditions are likely to be older and receiving more medications, which is associated with health care spending increases and low medication adherence.^19,20^ A total of 37% or more of US adults aged 62 to 85 years were estimated to be taking 5 or more medications.^21^ Comprehensive drug plans and benefits for the elderly people (including Medicare Part D and Advantage) may help alleviate the cost burden. In our subgroup analysis, elderly US people with Medicare coverage were only 50% to 80% more likely to buy their prescription drugs from other countries compared with those with additional private coverage or Medicare Part D benefits. Our study also found an association between internet use behaviors (seeking health information and online prescriptions) and medication purchases outside the US. Higher educational attainment is highly correlated with health information technology use (eg, patient portal).^22^ We observed a similar pattern between advanced academic degrees and medication purchases outside the US in this study. Thus, it is plausible that frequent use of internet and online resources among those with advanced degrees may be a factor associated with an increased odds of medication purchases outside the US. However, more studies are needed to elucidate the reasons why those with higher educational attainment chose to purchase medications outside the US.

Our findings have several implications for patients and their caregivers who have purchased drugs outside the US or who have been considering importing prescription medications to reduce their out-of-pocket cost burden. There are several risks and safety issues associated with purchasing of medications outside the US regarding counterfeit products and regulatory compliance with good manufacturing practices. Counterfeit medications, also known by the new term *substandard and falsified medications* adopted by the World Health Association, are associated with significant risks to patients in other countries and US citizens purchasing these products.^23^ The evidence suggests that more than 10% of all medications sold in the world are counterfeit, and the estimate is a high as 70% for some areas in Africa and Asia.^24,25^ These substandard medications have therapeutic concentrations that, in the case of antibiotics, could lead to development of antibiotic resistance in addition to the presence of other ingredients and contaminants besides the active ingredient.^23,26^ Manufacturing of medications in some countries might not be as strictly regulated as in higher-income countries, which raises safety concerns in particular for the production of generic products that might not meet good manufacturing practices or bioequivalence standards.^26,27^ Although the public is free to purchase medications outside the US, patients should be informed of potential risks they can encounter when using rogue internet pharmacies. These online pharmacies have a strong presence through social media posts, online chats and forums, and internet searches.^28,29^ Policies that seek to pursue drug importation from countries other than the US should consider the potential for drug manufacturing quality issues.

### Limitations

This study has limitations. First, we were able to estimate recent purchases, but we were not able to estimate purchasing by medication class (eg, antibiotics, cancer treatments, and erectile dysfunction medications). This information would be beneficial to provide context for which conditions patients believe they need to purchase medications outside the US. Previous evidence suggests that given the high costs of certain classes of drugs, cost-saving practices might be more prevalent for those patients.^30^ Second, the NHIS question on purchasing medications outside the US is primed to indicate that the main purpose for this activity is to save money. We were not able to capture other reasons for prescription purchase outside the US with the current data. Third, we only included information for patients who had complete information for variables of interest in this study. The percentage of survey respondents with missing information was low (<1.1%), and we do not expect that results would be significantly different for these patients. Fourth, this study did not address the source country of the medication. Previous literature has suggested that purchasing prescription medications without a prescription in a country outside the US or over the internet (eg, antibiotics) is also associated with risk^23,31^; however, this was not captured in the questions provided in the NHIS. Individuals living in states that neighbor Canada and Mexico are likely to go across the respective borders to purchase medications in the other country.^10,31,32^ The NHIS data only contain information on the US geographic region. However, individuals can purchase medications from Canada over the internet regardless of how close their residence is to Canada.

## Conclusions

The current study provides, to our knowledge, the first national benchmark estimates on the prevalence of purchasing of prescription medications by US adults in another country to save money. Among US adults taking prescription medications, 1.5% (representing 2.3 million US individuals) reported that they purchased their medications from countries outside the US to save money. Financial constraints on refilling prescription, along with inadequate coverage and internet use for health care, were significantly associated with medication purchase outside the US. Given the expected changes in prescription drug importation rules, continued monitoring appears to be needed to examine the patterns of medication purchase behaviors and factors that facilitate or impede the use of imported drugs. Future research regarding the economic evaluation of changing drug importation rules in the US health care system is also warranted.

## Notes

Supplement. **eTable 1.** Analytic Cohort Selection: National Health Interview Survey (NHIS) 2015-2017 **eTable 2.** Estimated Prevalence of Medication Purchases Abroad among US Adults on Prescription Medications, by Selected Subgroups
