# Supplementary material for: Socioeconomic and Demographic Characteristics of US Adults Who Purchase Prescription Drugs From Other Countries
Source: JAMA Netw Open. 2020 Jun 24;3(6):e208968. doi: 10.1001/jamanetworkopen.2020.8968 (PMC7315289; doi:10.1001/jamanetworkopen.2020.8968)
Supplement: Supplement. — eTable 1. Analytic Cohort Selection: National Health Interview Survey (NHIS) 2015-2017 eTable 2. Estimated Prevalence of Medication Purchases Abroad among US Adults on Prescription Medications, by Selected Subgroups [file jamanetwopen-3-e208968-s001.pdf]

Supplementary Online Content

Hong Y-R, Hincapie-Castillo JM, Xie Z, Segal R, Mainous III AG. Socioeconomic and demographic characteristics of US adults who purchase prescription drugs from other countries. *JAMA Netw Open*. 2020;3(6):e208968. doi:10.1001/jamanetworkopen.2020.8968

eTable 1. Analytic Cohort Selection: National Health Interview Survey (NHIS) 2015-2017

eTable 2. Estimated Prevalence of Medication Purchases Abroad among US Adults on Prescription Medications, by Selected Subgroups

This supplementary material has been provided by the authors to give readers additional information about their work.

© 2020 Hong Y-R et al. *JAMA Network Open*.

eTable 1. Analytic Cohort Selection: National Health Interview Survey (NHIS) 2015-2017

		NHIS 2015-2017	
**Step**	**Criteria**	**Inclusion**	**Exclusion**
1	Individuals aged 18 or older responded NHIS 2015-2017	93442	
2	Those prescribed medication by doctor/health professional, past 12 months	61974	31468
3	Excluded if having missing value on medication behaviors	61922	52
4	Excluded if having missing value on education	61700	222
5	Excluded if having missing value on employment	61688	12
6	Excluded if having missing value on immigration status	61680	8
7	Excluded if having missing value on health insurance	61524	156
8	Excluded if having missing value on health conditions/comorbidities	61499	25
9	Excluded if having missing value on health information use	61238	261

Final cohort

Sample size= 61,238

Estimated population size= 152,249,180

© 2020 Hong Y-R et al. *JAMA Network Open*.

eTable 2. Estimated Prevalence of Medication Purchases Abroad among US Adults on Prescription Medications, by Selected Subgroups

Characteristics	Unweighted No. / Total No. (%)	Weighted No. / Total No. (%) [a]	Adjusted Weighted Prevalence, % (95% CI) [b]	P-value
**Race/Ethnicity and Immigration Status**				
US-Born				<.001
NH-White	493 / 42628 (1.1)	1102009 / 102755952 (1.1)	1.5 (1.2-1.8)	
NH-Black	30 / 6452 (0.5)	62973 / 15249252 (0.4)	0.6 (0.4-0.9)	
Hispanic	65 / 3072 (2.1)	176382 / 8697302 (2.0)	2.9 (1.7-4.8)	
Other	5 / 1519 (0.3)	14567 / 3165211 (0.5)	0.6 (0.2-1.9)	
Immigrants				<.001
NH-White	63 / 1789 (3.5)	189775 / 4996138 (3.8)	4.7 (3.3-6.6)	
NH-Black	10 / 629 (1.6)	32440 / 2058527 (1.6)	1.9 (0.9-3.9)	
Hispanic	211 / 3342 (6.3)	597456 / 9705053 (6.2)	6.4 (4.9-8.3)	
Other	50 / 1807 (2.8)	164297 / 5621744 (2.9)	3.5 (2.3-5.2)	
**Age and Type of Insurance**				
18-64				<.001
Employment-Based	334 / 27495 (1.2)	952084 / 78593984 (1.2)	1.5 (1.2-1.9)	
Market Exchange	17 / 559 (3.0)	50141 / 1477128 (3.4)	3.4 (1.8-6.3)	
Medicaid or Other Public	102 / 9252 (1.1)	296916 / 23385302 (1.3)	1.1 (0.8-1.6)	
Uninsured	158 / 3172 (5.0)	389761 / 8232784 (4.7)	4.5 (3.4-6.0)	
≥65				.03
Medicare+Private	99 / 7396 (1.3)	204519 / 14755296 (1.4)	1.4 (0.9-2.0)	
Medicare+Public	22 / 2159 (1.0)	42996 / 3952618 (1.1)	0.9 (0.5-1.8)	
Medicare Only	48 / 1544 (3.1)	104359 / 2913961 (3.6)	3.7 (2.4-5.8)	
Medicare+Private+Part D	97 / 6528 (1.5)	211636 / 13115429 (1.6)	1.6 (1.1-2.3)	
Medicare+Public+Part D	16 / 1252 (1.3)	31761 / 2188645 (1.5)	1.2 (0.6-2.4)	
Medicare+Part D	34 / 1881 (1.8)	55726 / 3634031 (1.5)	1.6 (0.9-2.6)	
**Selected Chronic Conditions**				
Hypertension	413 / 28669 (1.4)	1041846 / 65498850 (1.6)	2.5 (1.9-3.4)	
Diabetes	139 / 9233 (1.5)	379452 / 21199116 (1.8)	2.5 (1.8-3.6)	
CVD	193 / 12344 (1.6)	443159 / 27111973 (1.6)	2.6 (1.9-3.6)	
COPD	29 / 1569 (1.8)	60055 / 3196570 (1.9)	2.9 (1.8-4.8)	
Cancer	145 / 8693 (1.7)	316302 / 19194975 (1.6)	2.7 (1.9-3.9)	

Abbreviations: NH, non-Hispanic; CVD, cardiovascular disease; COPD, chronic obstructive pulmonary disease

a Estimates are weighted to be nationally representative.

b Adjustments include age, sex, race/ethnicity, immigration, marital, and employment status, education, family income level, census region, type of insurance, self-reported health status, and the number of comorbidities.

© 2020 Hong Y-R et al. *JAMA Network Open*.
